# Update on the Relationship Between Depression and Neuroendocrine Metabolism

**DOI:** 10.3389/fnins.2021.728810

**Published:** 2021-08-31

**Authors:** Wenxin Qiu, Xiaodan Cai, Chenhui Zheng, Shumin Qiu, Hanyang Ke, Yinqiong Huang

**Affiliations:** ^1^Fujian Medical University, Fuzhou, Fujian, China; ^2^Department of Endocrinology, The Second Affiliated Hospital of Fujian Medical University, Quanzhou, China

**Keywords:** depression, metabolic syndrome, major depressive disorder, inflammation, endocrine

## Abstract

Through the past decade of research, the correlation between depression and metabolic diseases has been noticed. More and more studies have confirmed that depression is comorbid with a variety of metabolic diseases, such as obesity, diabetes, metabolic syndrome and so on. Studies showed that the underlying mechanisms of both depression and metabolic diseases include chronic inflammatory state, which is significantly related to the severity. In addition, they also involve endocrine, immune systems. At present, the effects of clinical treatments of depression is limited. Therefore, exploring the co-disease mechanism of depression and metabolic diseases is helpful to find a new clinical therapeutic intervention strategy. Herein, focusing on the relationship between depression and metabolic diseases, this manuscript aims to provide an overview of the comorbidity of depression and metabolic.

## Introduction

Depression is a kind of mental disorder with high prevalence rate, high clinical cure rate, but high recurrence rate, and its suicide rate is generally higher than that of the general population, which has become a major disease threatening the mental health of the public (Miret et al., 2013). According to the WHO, there are more than 300 million people with depression in the world, accounting for 4.4% of the world’s population. In 2020, depression will become the second largest human health burden after cardiovascular disease, with an economic burden of about $2.5 trillion, accounting for 10% of the total global disease burden (Liu et al., 2015; Tran et al., 2020).

In recent years, with the in-depth study of the mechanism of depression, there is evidence that depression is closely related to metabolic diseases, such as obesity, hypertension, diabetes, metabolic syndrome (MS; Ali et al., 2006; González and Tarraf, 2013; Euesden et al., 2017). For example, depression increases the risk of high blood pressure (Meng et al., 2012). Patients with type 2 diabetes (T2D) were 1.2–2.3 times more likely to have depressive symptoms than the general population (Arroyo et al., 2004; Knol et al., 2006; Mezuk et al., 2008; Mommersteeg et al., 2013). Studies have shown that obesity increases the risk of depression, and vice versa, and so does the risk of depression followed by obesity (Blaine, 2008; Luppino et al., 2010; Mannan et al.,2016a,b). However, compared with normal people, obese people with good metabolism have only a slightly increased risk of depression, but when obese people are accompanied by metabolic disorders (e.g., hypertension, dyslipidemia, high C-reactive protein or insulin resistance), the risk of depression is higher (Milaneschi et al., 2019). The relationship between obesity and depression may be related to neuroimmune pathway (Martins et al., 2019). Adipocyte hypertrophy can stimulate the production of inflammatory mediators (Gregor and Hotamisligil, 2011), thus affecting the central nervous system, which can cause neuroinflammation in the hypothalamus and hippocampus (Castanon et al., 2015). Studies have shown that psychological disorders and diabetes may exacerbate each other. Depression can inhibit the secretion of islet cells, thereby reducing the ability to regulate glucose metabolism in patients with diabetes (Vancassel et al., 2018), resulting in a high risk of death (Felger, 2017). In addition, the common disease diabetes will make the body in a state of chronic inflammation, depression also has a chronic low-grade inflammatory response. Patients with depression are often accompanied by elevated levels of proinflammatory cytokines; the level of peripheral inflammatory markers is related to the severity of depression; exogenous proinflammatory cytokines can induce depression; antidepressants can reduce the level of inflammatory markers in patients with depression to some extent (Bonaccorso et al., 2002; Suarez et al., 2003; Kim et al., 2007). These diseases extend from obesity to so-called “MS,” which leads to the activation of some inflammatory factors, cytokines and chemokines, such as interleukin-6 (IL-6) and C-reactive protein, which are significantly associated with MS and the severity of depression (McIntyre et al., 2007).

This article introduces the mechanisms of co-disease of depression and metabolic diseases, discusses the mechanism of neuroimmune-endocrine co-disease between depression and metabolic diseases, and summarizes the progress of comprehensive treatment of depression in recent years, which can help to explore the pathogenesis of depression, and find a new intervention stragegy for depression, and further reduce the incidence and recurrence rate of depression.

## Depression and Metabolic Disease

### Obesity

The association between obesity and depression may be related to neuroimmune pathways (Martins et al., 2019). In obesity, adipocyte hypertrophy triggers the recruitment of innate immune cells and stimulates the production of inflammatory mediators (Gregor and Hotamisligil, 2011). These cytokines then reach the central nervous system, especially in the hypothalamus and hippocampus (Castanon et al., 2015), triggering neuroinflammation. In today’s society, people who are obese may feel inferiority, leading to depressive symptoms, as thinness is considered the standard of beauty. Symptoms of depression, such as being sedentary and eating too much, may in turn contribute to obesity. During stress or depression, the appetite changes accordingly. For example, one study among college students found that depressive symptoms were positively associated with consumption of unhealthy foods, including candy, cookies, snacks, and fast food (El Ansari et al., 2014). Mood disorders can also lead to carbohydrate cravings and increased intake of sweet or starchy foods (Corsica and Spring, 2008; Ventura et al., 2014). Because foods with a high carbohydrate content can temporarily improve mood, especially when eating highly palatable foods activates the brain’s opioid system and produces a pleasure response (Ventura et al., 2014). In addition, most patients with depression are accompanied by symptoms of insomnia. Sleep is a major regulator of neuroendocrine function, and sleep disorders are associated with disturbed glucose metabolism, increased levels of ghrelin (appetite stimulating hormone), decreased levels of leptin (appetite stimulating hormone), and increased blood-brain barrier. These neurobiological mechanisms increase the risk of obesity in patients with depression and are related to the duration of depressive episode (Goldstein et al., 2011; Tully et al., 2020). It’s worth emphasizing that many antidepressants (such as mirtazapine and tricyclic antidepressants) and mood stabilizers, primarily valproate and lithium, have been associated with weight gain. According to recent studies, different degrees of weight gain may occur during the second and third years of antidepressant use (Gafoor et al., 2018).

**FIGURE 1:**
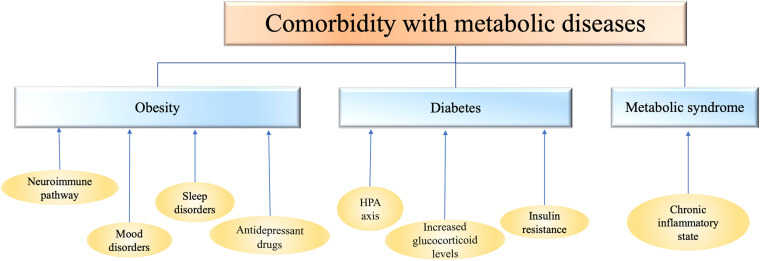
Depression is comorbid with metabolic diseases such as obesity, diabetes, and metabolic syndrome. The mechanism of comorbidity is closely related to the abnormalities of inflammation, endocrine system, nervous system and immune system. HPA, hypothalamic-pituitary-adrenal axis.

However, acute or unpredictable stressors lead to significant reductions in weight and food intake, while chronic social or predictable stressors lead to increases in calorie intake and weight gain (Howard et al., 1996; Pong et al., 1996; Kojima et al., 1999; Tschöp et al., 2000; Nakazato et al., 2001; Abizaid, 2019). Acute psychological or systemic stressors can also lead to rapid declines in food intake and utilization of carbohydrate stores (Howard et al., 1996). Some people in the face of acute or unpredictable stressors, there may be loss of appetite, sleep disorders, such as performance, resulting in food intake and weight loss. In addition, unhealthy weight loss strategy, also may lead to the emergence of depressive symptoms, at least use an unhealthy weight loss strategy related to report a 47% increased risk of depression (Chaitoff et al., 2019) (Figure 1).

### Diabetes

Patients with diabetes have been in a high glucose environment for a long time, and their bodies are in a chronic inflammatory state. Patients with T2D are generally accompanied by increased inflammatory factors, and a large number of evidences have shown that immune cells *in vitro* experiment appear inflammatory response in solution with high blood glucose concentration (Prattichizzo et al., 2018). By controlling fasting blood glucose, reducing fasting insulin level and improving insulin resistance, tumor necrosis factor (TNF) and IL-6 levels *in vivo* can be significantly reduced, and inflammatory state *in vivo* can be significantly improved (Borst, 2004). Depression is also associated with chronic low-grade inflammation. The level of proinflammatory cytokines is often increased in patients with depression. The level of peripheral inflammatory markers was correlated with the severity of depression. Exogenous proinflammatory cytokines can induce depression. Antidepressants can reduce the level of inflammatory markers in patients with depression to a certain extent (Bonaccorso et al., 2002; Suarez et al., 2003; Kim et al., 2007).

Although the mechanism of comorbidities between diabetes and depression is still unclear, we speculate based on some available evidence that the comorbidities between diabetes and depression may be related to the activation of certain inflammatory pathways, such as NLRP3 (NOD-like receptor protein 3) inflammatory bodies, which can accept the stimulation of metabolic stress signals and further cause the activation of Caspase-1 and a series of inflammatory factors. For example, interleukin-1 β (IL-1β) and interleukin-18 (IL-18) are continuously increased and their receptor expression is up-regulated under chronic hyperglycemia, which can lead to pancreatic β cell apoptosis, functional decline and insulin resistance. Il-1 β and other inflammatory factors such as TNF synergistically interfere with the balance of glucose metabolism *in vivo* (Zhao et al., 2014). The study (Zhang et al., 2014) of lipopolysaccharide induced depression-like behavior in mice found that NLRP3 inflammasomes inhibitor could block depressive behavior in mice. In addition, in diabetic patients with concurrent depressive symptoms, the inflammation in the body significantly increased (Shenhar-Tsarfaty et al., 2016) (Figure 1).

### Metabolic Syndrome

Metabolic syndrome is a global epidemic of multiple metabolic risk factors including obesity, insulin resistance, dyslipidemia and hypertension (Martins et al., 2019). Studies have shown that in patients with MS comorbidities depression, the function of the hypothalamic-pituitary-adrenal axis (HPA) is dysregulated (Southwick et al., 2005). Chronic increases in glucocorticoid levels, which block insulin’s ability to promote glucose uptake by cells, can lead to fat accumulation in the body. Long-term and serious accumulation of free fatty acids in the body will change insulin sensitivity (Sarafidis and Bakris, 2007), leading to obesity and diabetes. Secondly, insulin resistance is also one of the important symptoms of MS. The appearance of insulin resistance may damage neuroglial cells or neurons, causing changes in the body’s emotional state. For example, at the same insulin level, patients with insulin resistance have less cortical activation compared with normal individuals (McIntyre et al., 2007). In addition, the continuous high insulin level of the body can promote cell growth, lead to hyperplasia and hypertrophy of arterial wall, and aggravate hypertension and heart disease (McIntyre et al., 2007). In addition, the activation of some inflammatory factors, cytokines and chemokines in patients with MS is also closely related to the occurrence of depression. For example, studies have found that the concentrations of IL-6 and C-reactive protein are increased in serum or plasma of depressed individuals, and are significantly correlated with the severity of depression (McIntyre et al., 2007) (Figure 1).

## The Underlying Mechanism of Depression

### Inflammation

In recent years, many animal experiments and clinical studies have pointed out that the pathogenesis of depression is highly related to chronic inflammation. For example, some rodent research reports (Su et al., 2017; Han et al., 2018; Zhao J. et al., 2019; Ruilian et al., 2021) showed that central and peripheral concentrations of inflammatory factors, especially IL-1 β, IL-6, and TNF-α, increased after the depression model was established by chronic unpredictable mild stress (CUMS). Willner et al.’s (2013) study on major depressive disorder (MDD) found that mild depressive symptoms (such as depression, mental anxiety, and guilt) were associated with anti-inflammatory responses (high levels of IL-4 and low levels of IL-17 and IL-2). On the contrary, the clinical manifestations of severe depression, like psychomotor retardation, are associated with proinflammatory response, that is, high level of IL-6.

Experiments (Feng et al., 2019) have shown that NLRP3 inflammatory bodies can mediate hippocampal neuroinflammation and depression-like behavior induced by chronic stress through GR-NF-κ B-NLRP3 signal pathway. At the same time, it can lead to changes in the levels of hormones, mediators and inflammatory factors in endocrine regulation, and abnormal function or expression of some receptors. NLRP3 inflammatory bodies are widely involved in the pathophysiological process of MDD and are the current target for the treatment of MDD (Hyvärinen et al., 2019).

NOD-like receptor protein 3 is expressed in microglia and mediates hippocampal neuroinflammation and depression-like behavior induced by chronic stress through GR-NF-κB-NLRP3 signal pathway. The function of HPA axis is mediated by glucocorticoid receptor (GR), and GR is affected by epigenetic mechanism (DNA methylation). By using peripheral blood to detect the independent and longitudinal effects of methylation of three CpG sites in exon 1F of NR3C1 gene on senile depression, it was found that methylation of exon 1F of NR3C1, especially CpG2, was associated with senile depression (Kang et al., 2018). In addition, recent large randomized trials have shown that targeted inflammatory cytokine therapy can more effectively reduce depressive symptoms of inflammatory somatic diseases with MDD, such as psoriasis, inflammatory bowel disease and rheumatoid arthritis, compared with other treatments (Gordon et al., 2018; Strober et al., 2018). Other drugs with anti-inflammatory effects, such as selective serotonin reuptake inhibitors (SSRIs) and norepinephrine reuptake inhibitors, can regulate neural inflammation, including reducing blood or tissue inflammatory factors and regulating complex inflammatory pathways, thus achieving antidepressant effects (Dionisie et al., 2021). In the rodent depression model, the olfactory bulb resection rat model provides a more representative MDD model, which not only proved the increase of tissue proinflammatory cytokines, prostaglandin E2 and NO, but also showed that the symptoms of depression are alleviated after long-term anti-inflammatory treatment (Song and Leonard, 2005).

Elevated levels of inflammatory factors can also lead to abnormalities in the nervous system. Inflammatory factors affect the mechanism of central nervous system and neurotransmitter signal transduction pathway to produce depressive symptoms, which is an important part of the inflammatory pathogenesis of depression. This involves a series of complex mechanisms, including alteration of the monoamine system, hypothalamus-pituitary-adrenal axis, growth factor, neuropeptide and glutamate transmission, and reduction of nerve remodeling (Felger and Lotrich, 2013). Severe depression is associated with neurodegenerative changes associated with chronic inflammation. The function of nerve repair performed by neurotrophic factors is blocked, such as brain-derived neurotrophic factor (BDNF), then, the integrity of nerve cell membrane is affected, which leads to the repair of damaged dendrites, axons and axons is reduced (Lucassen et al., 2010). Studies have shown that some inflammatory factors, such as IL-1β, reduce hippocampal neurogenesis. In addition, there is evidence that the end products of the inflammation-activated tryptophan-canine pathway, such as 3-hydroxycanine and quinolinic acid, also play an important role in neurodegenerative changes in chronic severe depression (Goshen et al., 2008; Zunszain et al., 2012).

**FIGURE 2:**
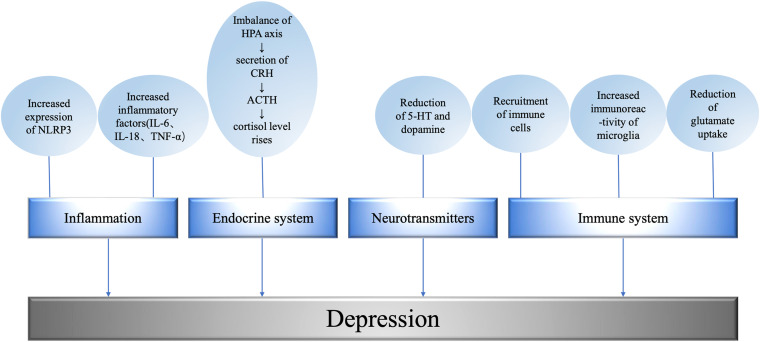
Mechanism underlying metabolism related depression. The level of promoting factors is related to the degree of depression; the imbalance of HPA axis leads to the abnormality of endocrine system; excitatory neurotransmitters regulate depressive behavior; the immune system affects the occurrence of depression at the cellular and molecular level. NLRP3, NOD-like receptor protein 3; IL, interleukin; TNF, tumor necrosis factor; HPA, hypothalamic-pituitary-adrenal axis; CRH, corticotropin releasing hormone; ACTH, adrenocorticotropic hormone; 5-HT, 5-hydroxytryptamine.

Therefore, the incidence of depression may be closely related to chronic neuroendocrine immune inflammation (Figure 2).

### Endocrine System

In the endocrine regulation of patients with depression, the levels of hormones, mediators, and inflammatory factors all change, and the function or expression of some receptors are abnormal. For example, stress and other factors will lead to HPA axis imbalance, leading to the secretion of corticotropin releasing hormone (CRH), CRH stimulates adrenocorticotropic hormone (ACTH). ACTH then stimulates the adrenal gland to release cortisol, causing cortisol levels to rise (Golden et al., 2011). It has been found that overexpression of angiotensin converting enzyme 2 (ACE2) in corticotropin-releasing hormone, CRH cells can inhibit stress-induced activation of HPA axis and down-regulate CRH synthesis in paraventricular nucleus and central amygdaloid nucleus (CeA). The activation of passivated HPA axis was observed in CRHACE2KI mice as a result of increased ACE2 production. Overexpression of ACE2 in CRH cells did not affect the response of adrenal gland to adrenocorticotropin, ACTH. The overexpression of ACE2 is related to the transcription of CRH, which leads to a significant increase in the expression of ACE2mRNA in paraventricular nucleus and CeA. The increase of exogenous ACE2 inhibited the synthesis of CRH, while the decrease of CRH synthesis changed the central management of psychological stress, thus weakening the activation of HPA axis and alleviating anxiety-like behavior (Wang et al., 2018) (Figure 2).

### Neurotransmitters

Through the simultaneous determination of plasma tryptophan metabolites in healthy controls (HC) and MMD patients by ultra-high speed liquid chromatography/mass spectrometry, it was found that the plasma 5-HT level in MMD patients was significantly higher than that in HC (Tomioka et al., 2020). 5-HT1A receptors are widely distributed in the brain. Study (Richardson-Jones, 2009) found that 5-HT1A receptor has a regulatory effect on depressive behavior. When the expression of 5-HT1A receptor decreased, the depressive behavior increased; when the 5-HT1A receptor was activated and increased, the depressive behavior decreased.

By analyzing the slope amplitude of N1/P2 components of auditory evoked potentials and the determination of L-tryptophan free fraction in plasma, it was found that the brain activity of serum neurosin and L-tryptophan free fraction decreased in women with depression (Vázquez-Estupiñan et al., 2019). After ovariectomy in female mice, it was found that while estrogen decreased, the expression of IL-1β and IL-18, NLRP3 and the level of inflammatory cytokines such as active caspase-1 in the hippocampus of female mice increased significantly. This suggests that estrogen deficiency can lead to activation of NLRP3 inflammatory bodies, which leads to hippocampal nerve inflammation, depression and anxiety (Xu et al., 2016).

Inflammatory factors can affect the synthesis of neurotransmitters, more accurately, destroy the production of 5-HT and dopamine, and down-regulate tetrahydrobiopterin (BH4) through reactive oxygen species and reactive nitrogen species, and then inhibit the synthesis of norepinephrine. BH4 is the key cofactor of tryptophan hydroxylase and tyrosine hydroxylase, which happen to be necessary for tryptophan and tyrosine to synthesize 5-HT and dopamine (Felger and Lotrich, 2013; Vancassel et al., 2018). And studies have found that inflammatory factors affect dopamine secretion and reward circuits, leading to depressive symptoms such as lack of pleasure, fatigue and psychomotor retardation (Felger, 2017). In the experiment of single injection of lipopolysaccharide (LPS) (0.5 mg/kg, I.P.) and unpredictable chronic mild-stress (UCMS) for 4 weeks, it was found that compared with UCMS stressed mice, LPS stressed mice had longer fixed time in forced swimming test and tail suspension test, and longer time around open field test. In addition, compared with UCMS stress mice, LPS stress mice showed stronger expression and release of TNF-α, IL-1 β, and IL-6 in serum and depression-related brain regions (frontal cortex, hippocampus, and striatum). The results of enzyme linked immunosorbent assay showed that compared with UCMS, LPS stress induced peripheral and central immune activation, increased expression of indoleamine 2, 3-dioxygenase and more severe depression-like behavior in rats (Zhao X. et al., 2019). The level of plasma epidermal growth factor in patients with MDD and HC was significantly lower than that in patients with HC (Tian et al., 2012). The levels of FGF-2 protein and central FGFR1 RNA in peripheral blood of patients with MDD were significantly higher than those of HC (Wu et al., 2016). Vitamin D normally functions to maintain low intracellular Ca^2+^ levels, but when vitamin D levels decrease, Ca^2+^ levels begin to rise in the cells, which may exacerbate the onset of depression. In addition, vitamin D plays a role in reducing inflammation, maintaining 5-HT synthesis and inducing the expression of DNA demethylase that controls the epigenetic environment, thereby maintaining normal neuronal activity in gene transcription and preventing depression (Berridge, 2017). The level of insulin-like growth factor (IGF)-1 in peripheral blood of patients with MDD was significantly higher than that of HC, and negatively correlated with the duration of the disease (Tu et al., 2016). However, compared with HC, patients with depression have lower serum and plasma BDNF concentrations. Serum BDNF and IGF-1 levels may be a potential combination of biomarkers and can be used as diagnostic tests for MDD (Troyan and Levada, 2020). The severity of clinical symptoms was assessed by Hamilton Depression scale (HAMD-17) and Hamilton anxiety scale (HAMA-17) in patients with MDD and HC, and the differences of glial cell line-derived neurotrophic factor (GDNF) levels among different subgroups were compared. It was found that the level of protective neurotrophic GDNF in the brain of patients with MDD was related to age (Sun et al., 2019). The protein and mRNA expression of GDNF decreased in patients with depression (Zhang Y. et al., 2017). The level of GDNF in patients with advanced MDD was significantly decreased (Diniz et al., 2012). In patients with MDD, the functional connectivity of bilateral AFC networks [including bilateral insular lobe, hippocampus, temporal pole (TP), supramarginal gyrus (SMG), dorsomedial prefrontal lobe (DmPFC), left fusiform area (FFA), inferior temporal gyrus (ITG), inferior parietal lobule (IPL), and parahippocampal gyrus (PHG)] was generally decreased, and even bilateral medial prefrontal connectivity changed from negative to positive (He et al., 2019).

The hypothalamus senses and integrates various signals from the blood and the third ventricle and regulates food intake (Kohno and Yada, 2012; Schneeberger et al., 2014). In addition, they receive neural signal inputs from multiple parts of the central nervous system (Dörr et al., 2015; Sohn, 2015). Studies have shown that hypothalamus can regulate the feeding process and subsequent material transformation through neuronal activation. For example, in the lateral hypothalamus, ghrelin may increase food intake, food reward, and arousal by stimulating orexin neurons (Abizaid, 2019). NPY/AgRP neurons and POMC neurons are a group of antagonistic neurons, which not only regulate the feeding process, but also participate in the coordination of subsequent carbohydrate and lipid conversion, storage and utilization (Varela and Horvath, 2012). NPY/AgRP signaling in adult mice is important for maintaining normal lipid and glucose homeostasis in peripheral tissues such as liver, muscle, and pancreas. The consumption of NPY/AgRP neurons in newborn mice affects the balance of lipid and carbohydrate metabolism. Mice lacking NPY neurons developed obesity and hyperinsulinemia after a normal diet (Huang et al., 2021).

In a randomized controlled trial of MBSR altering amygdala functional connectivity during weight loss maintenance, changes in the VENtral and medial prefrontal cortex FC were associated with changes in depressive symptoms, with significant FC interactions between the amygdala and the ventral and medial prefrontal cortex. FC increased in the MBSR group (mindfulness-based Stress Reduction), while decreased in the control group. At 6 months, the MBSR group was observed to maintain weight while the control group had a 3.4% increase in bmi (Chumachenko et al., 2021).

A study of hippocampus volume and depression severity in children with depression demonstrated for the first time that children with more severe depression experienced decreased hippocampal volume at the onset of depression, and that the reduction was associated with depression severity, while the reduction was not significant in children with less severe depression (Barch et al., 2019). Roddy et al. (2019) examined the hippocampal volume of 80 patients with MDD and 83 patients without MDD. Using advanced high-quality hippocampal segmentation technology, we performed automatic segmentation of hippocampal substructures in patients with first-episode depression and recurrent depression, and found that the left hippocampus of PATIENTS with MDD was smaller. Roddy et al. (2019) also believe that the hippocampal definition is necessary to reasonably explain future MDD findings, and hypothesize that the extent of hippocampal volume reduction in MDD patients depends on the hippocampal definition used, and that the more specific the hippocampal definition is, the greater the degree of hippocampal volume change in patients with depression (Roddy et al., 2019).

Activation of the sympathetic nerve, which determines the increase in blood pressure and heart rate, also suppresses the parasympathetic branch, which regulates the immune response through the afferent and efferent fibers of the vagus nerve, enabling it to prevent excessive inflammation (Ouakinin et al., 2018). Sympathetic activation also induces rapid glycogen decomposition and glucose production in the liver, and enhances lipolysis of adipose tissue, directing lipid substrates to gluconeogenesis, leading to elevated blood glucose. In addition, catecholamines from the adrenal medulla further increase hepatic glucose output (Carnagarin et al., 2018).

Chronic overactivity of the sympathetic nerve may lead to diabetes. Evidence from an 18-year follow-up study in young Norwegian men suggests that sympathetic hyperactivity precedes the development of prediabetes and insulin resistance (Flaa et al., 2008). In addition, long-term sustained sympathetic excitation also inactivates the postpranalateral increase in skeletal muscle blood flow, resulting in impaired glucose uptake, hyperinsulinemia, and insulin resistance (Julius and Valentini, 1998). These symptoms can lead to chronic inflammation in the body and contribute to depression (Figure 2).

### Immune System

As early as a century ago, Nobel laureate Julius Wagner-Galleg observed that mental states are associated with the activation of the immune system (Cruz-Pereira et al., 2020). With the development of psychoneuroimmunology, more and more studies have shown that the changes of various psychological parameters in patients with infection during the onset and duration are similar to those in patients with depression (Dantzer, 2018). On the one hand, the inflammatory response given by the body to infectious factors or cytokines can be transmitted to the central nervous system through inflammatory factors or immune cells, thereby inducing disease behaviors, namely infection-related behavioral changes, such as fatigue, anhedonia, and anorexia (Capuron and Miller, 2004; Dantzer et al., 2008). On the other hand, among the 72 psychosocial stressors, acute and chronic stressors can also transduce immune signals in the brain parenchyma, and regulate systemic immune activities and intra-brain and inter-regional communication, thus influencing psychological functions and behaviors (Iwata et al., 2013; Duman, 2014). In addition, more and more people are found to have an autoimmune disease of the individual risk of depression significantly increased (Maes et al., 1995), such as in COVID-19 survivors at the hospital in a month of follow-up after treatment in the study of psychopathology in the investigation found that because the immune response of coronavirus induction of cytokines and chemokines, and other local and systemic inflammatory mediators, and make them complicated with post-traumatic stress disorder, depression, anxiety, insomnia, and compulsive symptoms improved obviously (Mazza et al., 2020), and because many of the anti-inflammatory effect of antidepressant drugs, Neuroimmune mechanisms are now considered to be central to the development of depressive symptoms (Hashioka et al., 2007).

#### Cytokines

In the pathophysiology of depression, the most important role of the non-specific immune system is in the recruitment of immune cells through the production of cytokines, activation of complement cascades, and subsequent activation of the adaptive immune system through antigen presentation. A study found that the levels of immune cells such as monocytes and granulocytes were increased in patients with depression (Cruz-Pereira et al., 2020). Many studies have also demonstrated an increase in serum concentrations of immune signaling molecules (chemokines and adhesion molecules), soluble intracellular adhesion molecule-1 and E-selectin, as well as acute phase proteins and proinflammatory cytokines (e.g., IL-6 or proinflammatory cytokines, prostaglandin) in patients with depression, suggesting that the immune system is involved in depression (Miller and Raison, 2016). Multiple studies have shown that the production and increase of peripheral cytokines, especially pro-inflammatory cytokines, may play a role in the onset and maintenance of depression (Maes et al., 1992, 1997, 2011; Lanquillon et al., 2000; Dean et al., 2010; Dowlati et al., 2010; Fagundes et al., 2013). Proinflammatory cytokines, such as IL-1, interferon (IFN)-γ, TNF-α, etc., can lead to depression-like behavior and mood disorders by affecting synaptic plasticity (Tian et al., 2012). In rodents, depression-like behavior induced by the use of chronic social defeat stress, male sterility, and luteinizing hormone has been associated with higher levels of pro-inflammatory IL-1β, IL-6, and TNF-α (Grippo et al., 2005; Kubera et al., 2011; Wohleb et al., 2011; Hodes et al., 2014). In contrast, anti-inflammatory IL-10 was reduced in the cortex and hippocampus of rats subjected to chronic bondage stress, and depression-like behavior was reversed by administration of recombinant IL-10. In addition, activation of the immune system and increased production of pro-inflammatory cytokines affect multiple biological targets associated with depression (Voorhees et al., 2013), including cell proliferation, neurogenesis, gliogenesis and apoptosis (Kubera et al., 2011; Borsini et al., 2015). For example, chronic light deprivation also induces IL-6-dependent depression-like behavior through activation of nuclear factor-κB (NF-κB) signaling and may be associated with seasonal affective disorder marked by depressive symptoms (Monje et al., 2011).

#### Microglia

Microglia are key non-specific immune cells that exist in the central nervous system and monitor the environment by protrudes to determine changes in the physiological environment. In addition to playing a key role in response to infection and injury, microglia are also involved in neuronal changes throughout neural development and at different stages, including synaptic remodeling to form neural network signals, etc. (Norris and Kipnis, 2019). Cytokines produced in peripheral and central nervous system increases with the activation of microglia, microglia can release inflammatory cell factor to influence the neuronal activity and neurotransmitter receptors transshipment and gene expression, neurons by including chemokines, cytokines, and neurotransmitters (CX3CL1, TGF-β, CSF-1, UDP, ATP, glutamate and GABA, norepinephrine, NE), soluble factor to adjust the function of microglia, in order to promote the function of microglia and cellular adaptation, guiding the movement and phagocytosis, and starting and spread the appropriate inflammation (Wohleb, 2016). The interaction between glial cells and neurons may promote neuroplasticity, neurogenesis, proliferation, pruning, and neurodegeneration throughout the life cycle, and may play a key role in stress and neuroinflammatory responses, which are significantly associated with changes in synaptic plasticity, neurogenesis, and emotional behavior (Li and Barres, 2018). Higher microglial immune reactivity has been reported in the cortices of patients with depression (Steiner et al., 2011) and suicidal subjects (Steiner et al., 2008). In one study, activation of larger microglia in the insular lobe was shown during major depressive episodes, particularly in the prefrontal cortex, anterior cingulate cortex, and insula (Setiawan et al., 2015). In rats, chronic binding stress also affects the density and morphology of microglia in stress-sensitive brain regions (Tynan et al., 2010) and can be reversed with minocycline treatment (Hinwood et al., 2013). Repeated social failure is also associated with microglia hyperplasia in the hippocampus, prefrontal cortex, and amygdala of mice, as well as an increase in inflammatory markers on microglia and central nervous system macrophages (Wohleb et al., 2011).

Glutamate is an excitatory transmitter in the brain whose transmission and conversion are strictly regulated (Sen et al., 2021). Astrocytes use glutamine synthetase to convert glutamate to glutamine (Martinez-Hernandez et al., 1977), which is then supplied to neurons. Neurons convert glutamine to glutamate via glutaminase (Kvamme et al., 2000) and repackage it into synaptic vesicles (Fremeau et al., 2004) activating microglia to release glutamate via cystine/glutamate antiporter (Mesci et al., 2015; Kitagawa et al., 2019). At the same time, activated microglia also release pro-inflammatory cytokines, activate astrocytes (Bezzi et al., 2001; Liddelow et al., 2017), induce glutamate release from astrocytes (Casamenti et al., 1999; Bezzi et al., 2001), and interfere with glutamate uptake (Hu et al., 2000; Mandolesi et al., 2013; Hyvärinen et al., 2019). Proinflammatory cytokines reduce the expression of excitatory amino acid transporters (EAATs) in astrocytes (Korn et al., 2005; Sitcheran et al., 2005). For example, depression induced by CUMS is associated with decreased expression of glial EAATs in the hippocampus and cerebral cortex (Banasr et al., 2010; Martín-Hernández et al., 2019). Autopsy studies of MDD patients have also shown decreased expression of EAATS in the anterior cingulate cortex and dorsolateral prefrontal cortex (Choudary et al., 2005) and in the hippocampus (Medina et al., 2016) (Figure 2).

## Clinical Therapeutic Intervention Strategy

The treatment targeting depression include a variety of strategies. The goal of the treatment of depression is to improve the clinical cure rate as much as possible, prevent recurrence and improve the quality of life of patients. At present, the clinical treatment of depression includes pharmacological treatment and non-pharmacological treatment. Non-pharmacological therapy includes psychotherapy, physiotherapy, electroconvulsive therapy, transcranial magnetic stimulation, vagus nerve stimulation(VNS; Kucia et al., 2019), Sandplay therapy also known as Sand spiels and play technique, Painting therapy, Suitable techniques of traditional Chinese medicine and so on (Zhang X. et al., 2017).

### Pharmacological Treatment

At present, pharmacological therapy is the most important clinical treatment for depression, SSRIs is widely used in pharmacological therapy. Among them, serotonin drug Agomelatine significantly reduced the scores of depression and anxiety at the end of the treatment of depression caused by type 2 diabetic comorbidities (Norman and Olver, 2019). Anti-hyperglycemic such as Insulin and Anti-Hyperglycemic Agents, which agents have demonstrated antidepressant properties in clinical trials, probably due to their action on brain targets based on the shared pathophysiology of depression and T2DM (Woo et al., 2020). In addition, there are tricyclic antidepressants, monoamine oxidase inhibitor, atypical antidepressants, and some traditional Chinese medicine antidepressants (Zhang X. et al., 2017). Such as Chaihu Shugan Powder, compound Chai Gui Fang, Prince Shenyue capsule and Kaixin Powder and other drugs (Zhang X. et al., 2017). Curcumin is a plant alkaloid obtained from Curcuma longa with potent anti-inflammatory and antioxidant effects (Asadi et al., 2020). Curcumin has a very low absorption. Due to this reason, it is preferable to use nano-curcumin, which has far greater bioavailability than curcumin (Rahimi et al., 2016). Nano-curcumin has good effect on depression and anxietyin diabetic patients peripheral neuropathy. In addition, Silymarin (Camini and Costa, 2020), Phytochemicals (Asadi et al., 2020), prebiotics (Paiva et al., 2020) and so on can also play a therapeutic role. However, there are still some shortcomings in pharmacological therapy. According to research, the effective rate of antidepressant treatment for the first time is only about half, and the treatment effect for recurrent depression is lower, cannot effectively control the condition of patients, some patients cannot achieve satisfactory results through drug treatment. And drug treatment is generally slow to take effect, cannot quickly alleviate the symptoms of patients. A recent systematic review concluded that, if possible, SSRIs should be selected to treat depression in patients with diabetes. Agomelatine and bupropion may have some efficacy, but more evidence is needed to prove their effectiveness (Wu et al., 2016).

### Psychotherapy

When the patient’s condition is mild, non-drug therapy such as drug therapy combined with psychotherapy is used to treat the patient. psychotherapy is mainly by correcting the bad cognition of the patients in order to reduce their depression. at the same time, family and social collective support therapy is adopted to achieve curative effect, in order to eliminate the psychosocial problems of patients and help to improve the condition. It can also reduce or avoid adverse drug reactions (Malhi and Mann, 2018). Depression is one of the common diseases among teenagers, and the prevalence rate is increasing year by year. SPT treatment is one of the more common treatment methods, which can enhance their psychological resilience, promote patients to release repressed emotions and regulate emotions. Increase the sense of belonging and promote the development of patients’ realistic interpersonal relationship. In recent years, other alternative therapies are also widely used in treatment, such as electroconvulsive therapy, transcranial magnetic stimulation, deep brain stimulation, VNS and so on. The long-term effect of VNS therapy is considerable (Kucia et al., 2019). VNS can be considered for patients with long-term depression and limited drug treatment (Müller et al., 2018). It is mainly used in the Treatment-Resistant Depression, and its adverse reactions are relatively low (Kucia et al., 2019). Secondly, diet can be used to treat metabolic diseases and comorbidities with depression. For example, ketogenic diets have been effectively utilized to treat a range of neurological metabolic diseases and, more recently, mental illnesses. More recently, the ketogenic diet has been shown to be an effective treatment for obesity and type II diabetes, and evidence is emerging for its use in manifold neurological disorders (Cox et al., 2019). Saffron (*Crocus satious* L.) may relieve symptoms of mild to moderate CDA in T2D patients. Because of non-considerable side effects, saffron may be suggested as an alternative treatment for CDA in diabetic patients (Milajerdi et al., 2018).

But at this stage, all kinds of anti-depression drugs and therapeutic effects on the market cannot achieve the desired results. Most drugs take effect slowly, and have obvious adverse reactions, which are considered only unilaterally and are not comprehensive enough. The treatment of depression should be combined with psychological therapy and drugs at the same time, which can effectively improve the therapeutic effect of depression. Now we are constantly trying a variety of new treatments, such as computer cognitive function training therapy, mobile application therapy and so on. In a word, the treatment of depression needs to be combined with a variety of methods, and all kinds of treatments should be personalized for patients. There is still much room for development in the current medical treatment of depression.

## Conclusion

Based on the above discussion, it can be seen that the mechanism of depression is complex at both molecular level and individual level, and there is a certain correlation at all levels. Moreover, depression is co-morbid with a variety of metabolic diseases, such as T2D, obesity, and MS. Therefore, the study of depression should not be limited to depression itself. Due to the impact of depression on individuals and society, the research on depression is still in-depth, which is helpful to design novel treatment strategies. Patients with depression suffer from long-term depression, slow thinking, decreased consciousness, cognitive impairment, and even sleep disorders, fatigue, loss of appetite, weight loss and other physical symptoms, which seriously affect their life. What’s more, the consultation rate of depression lags far behind other diseases, more than 90% of patients cannot get effective diagnosis and treatment. Based on the current situation, if we can explore the pathogenesis of depression, it will play a great role in the diagnosis and treatment of depression. Therefore, more research is needed to provide a new strategy for the treatment of depression in the future.

## Author Contributions

WQ and XC contributed in literature search and manuscript writing. CZ, SQ, and HK made the figures and the manuscript writing. YH contributed in the manuscript structure design and manuscript writing. All authors read and approved the final manuscript.

## Conflict of Interest

The authors declare that the research was conducted in the absence of any commercial or financial relationships that could be construed as a potential conflict of interest.

## Publisher’s Note

All claims expressed in this article are solely those of the authors and do not necessarily represent those of their affiliated organizations, or those of the publisher, the editors and the reviewers. Any product that may be evaluated in this article, or claim that may be made by its manufacturer, is not guaranteed or endorsed by the publisher.

## Abbreviations

5-HT: 5-hydroxytryptamine
ACE2: angiotensin converting enzyme 2
ACTH: adrenocorticotropic hormone
BDNF: brain-derived neurotrophic factor
BH4: tetrahydrobiopterin
CeA: central amygdaloid nucleus
CRH: corticotropin releasing hormone
CUMS: chronic unpredictable mild stress
EAATs: excitatory amino acid transporters
GDNF: glial cell line-derived neurotrophic factor
GR: glucocorticoid receptor
HC: Health control
HPA: hypothalamic-pituitary-adrenal axis
IFN: interferon
IGF: insulin-like growth factor
IL: interleukin
LPS: lipopolysaccharide
MDD: major depressive disorder
MS: metabolic syndrome
NLRP3: NOD-like receptor protein 3
PVN: paraventricular nucleus
SSRIs: selective Serotonin Reuptake Inhibitors
TNF: tumor necrosis factor
UCMS: unpredictable chronic mild-stress model
VNS: vagus nerve stimulation.
